# Conventional vs. Sutureless Aortic Valve Bioprosthesis: Is Faster Better?

**DOI:** 10.3390/jcdd10070311

**Published:** 2023-07-20

**Authors:** Ali Aljalloud, Ajay Moza, Jessica Paola Arias, Matthias Menne, Michael Becker, Konstantina Spetsotaki

**Affiliations:** 1 Department of Thoracic and Cardiovascular Surgery, RWTH University Hospital Aachen, 52074 Aachen, Germany; amoza@ukaachen.de (A.M.); jessikariasp@hotmail.com (J.P.A.); 2 Department of Cardiology, Rhein Maas Klinikum, 52146 Würselen, Germany; michael.becker@rheinmaasklinikum.de; 3 Department of Cardiovascular Engineering, Institute of Applied Medical Engineering, Helmholtz Institute, Medical Faculty, RWTH Aachen University, 52074 Aachen, Germany; menne@ame.rwth-aachen.de; 4 Department of Thoracic Transplantations and Assist Devices, Cardiothoracic Surgery, University Hospital Essen, 45147 Essen, Germany; konstantina.spetsotaki@uk-essen.de

**Keywords:** perceval bioprosthesis, sutureless valve, conventional bioprosthesis

## Abstract

**Purpose**: The benefits of sutureless compared to conventional aortic valve prosthesis replacement remain controversial. Supposed advantages of sutureless aortic valve replacement include shortened cross-clamp and implantation time, as well as improved overall safety and good post-operative performance. We aimed to compare the early outcomes and performance of sutureless aortic valve replacement (su-AVR) with the sutureless Perceval (Corcym, Milan, Italy) vs. the conventional AVR with a conventional counterpart, in this case, the Labcor Dokimos Plus (LDP) aortic bioprosthesis. **Methods**: We compared two types of aortic valve prostheses, the sutureless (Corcym, Milan, Italy) and the conventional valve Labcor Dokimos Plus (LDP), implanted between August 2014 and May 2019 in our Department of Cardiac Surgery at RWTH Aachen University Hospital. Data were collected from 141 patients who received the Perceval (Corcym, Milan, Italy) and 138 who received the Labcor Dokimos Plus (LDP) aortic bioprosthesis. After matching the two groups considering STS mortality risk and pre-operative LDH levels, 201 patients were included in our final study cohort. Seventy-one patients (17 from the Perceval group and 54 from the Dokimos group) were excluded due to the lack of complete data, particularly standardized echocardiographic data (*n* = 71). Primary endpoints were 30-day mortality, length of hospital stay, and pacemaker implantation. Secondary endpoints were echocardiographic parameters, major adverse cardiovascular events, and prosthesis failure (grade II aortic regurgitation, paravalvular leak with reintervention). **Results**: Bypass and cross-clamp time proved to be shorter in the Perceval group, while hospital stays were longer. The faster implantation had no effect on the 30-day mortality primary endpoint. Transvalvular gradients were significantly higher in the Perceval group, in addition to a smaller effective orifice area. The LDH values were remarkably higher post-operatively in the Perceval group. **Conclusions**: Regarding the clinical outcomes, Perceval was equivalent and not superior to the Dokimus bioprosthesis. The suitability of a Perceval prosthesis implantation must be determined on a case-by-case basis and reserved for elderly patients with increased comorbidity.

## 1. Introduction

Due to gradually increasing age expectancies, aortic valve stenosis has become the most common valvular disease in developed countries [1,2]. The onset of symptoms of which predicts a substantially reduced life expectancy. The lethality of untreated severe aortic valve stenosis is extremely high, with over 75% of patients dying within three years’ time after symptom onset. Severe and symptomatic aortic stenosis is related to poor outcomes without aortic valve replacement [3,4]. 

Aortic valve replacement, either via a surgical or percutaneous approach, remains the standard of care for severe aortic valve stenosis and is widely viewed as a safe, effective, and time-proven technique with both excellent short and long-term results [5,6]. 

Due to the aforementioned increasing age and resulting multiple comorbidities of the general population and the increase of combined operations, especially with coronary artery bypass grafting, various surgical alternatives to the conventional aortic valve replacement (c-AVR), such as the sutureless aortic valve replacement (Su-AVR) through conventional sternotomy or minimal surgical approaches, have been introduced [7,8]. 

Su-AVR is an attractive alternative for elderly patients with high risk who did not meet the conditions for transcatheter aortic valve replacement (TAVI).

Su-AVR, also known as the rapid valve deployment technique, is an alternative to the sutured prosthesis and enables the removal of the calcified valve, the fast deployment under direct supervision while minimizing the need for suturing, which consequently reduces the cardiopulmonary bypass (CPB), aortic cross-clamp, and operative time (ACC) [9,10,11]. Many previous studies report a strong relation between prolonged CPB time and increased morbidity and mortality in high- and low-risk patients [12,13,14]. Because of these potential benefits, the Su-AVR technique has gained ground in the last decades, especially in elderly, high-risk patients and in technically demanding procedures (e.g., minimally invasive surgery, calcified aortic root, combined cardiac procedures, and reoperations) [15]. The Perceval prosthesis is currently one of the most widely used sutureless prosthesis in the world [7].

The sutureless valve is especially designed with bovine pericardium leaflets while lacking a sewing ring to provide a larger EROA leading to improved hemodynamics when compared to other valve prostheses [7]. Although multiple studies have reported favorable short- and mid-term hemodynamic characteristics [8,16,17], several recent publications have brought major shortcomings of the Perceval prosthesis to light [18,19].

The purpose of this study is to compare the clinical outcomes and hemodynamic performance characteristics of c-AVR using the supra-annular stented pericardial Dokimos Plus valve bioprosthesis (Labcor, Belo Horizonte, Brazil) to the Su-AVR using the Perceval (Corcym, Milan, Italy) sutureless valve bioprosthesis. 

## 2. Material and Methods

### 2.1. Data Source

Patient data, including their demographics, intraoperative, and post-operative clinical results, were retrospectively collected using the database of our institution. The local ethics board approved our study (Ethics Commission RWTH Aachen, IRBP 10/2014, and EK 151/09-Version-1.3) and waived informed consent due to the study’s retrospective nature. 

### 2.2. Study Cohort

In this single-center, retrospective study, clinical, operative, and echocardiographic data of patients who underwent isolated elective surgical aortic valve replacement AVR with either Perceval or Dokimus bioprosthesis were collected and compared. Data on patients who received a surgical AVR in our department between August 2014 and May 2019 were obtained from the institutional database and subsequently screened. Inclusion parameters were elective cases of isolated AVR with either the sutureless Perceval or Dokimus conventional bioprosthesis. Exclusion criteria included emergent or urgent cases, patients with a bicuspid aortic valve, patients with anatomic contraindications to Su-AVR, such as aortic root dilation, endocarditis, hypertrophic cardiomyopathy with obstruction of LV outflow tract, re-do cases, inaccurate TTE quality post-operatively or lack of sufficient data. 

A total number of 279 patients underwent a surgical aortic valve replacement (SAVR) with Perceval and Dokimos in the Department of Cardiac Surgery at the RWTH Aachen University Hospital during the period from August 2014 to May 2019. Of these, 141 patients received Perceval, while 138 received the Labcor Dokimos. The aforementioned numbers solely reflect the number of patients who received one of the two valves in question and are not reflective of the total amount of aortic valve replacement surgeries that were conducted at our department during this time. 

The Labcor Dokimos Plus (LDP) aortic bioprosthesis Is a stented bovine pericardial bioprosthesis characterized by the externally mounted leaflet design, which results in low-profile blood flow. This minimizes leaflet and commissural stress, improving its effective orifice area (EOA) and leaflet movement, while reducing the risk of leaflet abrasion, stent deformation, and calcification. It allows for both intraanular and supraannular implantation and has achieved satisfactory performance and clinical outcomes comparable to other stented pericardial substitutes [20].

The Perceval prosthesis, on the other hand, comprises a bovine pericardium-formed leaflet attached to a nitinol stent. In preparation for implantation, the prosthesis is collapsed using a Perceval Collapser device, then it is released to take on its original shape after proper positioning over guide sutures. After the stent is deployed, post-dilation is performed with the assistance of a balloon to optimize the contact of the stent with the annulus [7].

### 2.3. Echocardiographic Analysis 

All patients underwent a standardized pre-operative and post-operative transthoracic echocardiogram (TTE) according to the American Society of Echocardiography (ASE) and the European Association of Cardiovascular Imaging (EACVI) [21]. Pre-discharge TTE was obtained in all patients during the 7 ± 2 post-operative days (POD). Patients were examined in the left lateral decubitus position with standard 2D images, including three cardiac cycles saved in cine-loop digital form for offline analysis. All echocardiography analyses were performed using the Vivid E9 (GE Vingmed Ultrasound AS, Horton, Norway) and the measurements were assessed with the EchoPAC version BT 202 (GE Vingmed Ultrasound AS). Complete offline analyses of the valve and LV performance were accordingly performed by an expert. These included gradients analysis, M-mode, 2D and tissue-Doppler imaging [4], as well as 2D-STE. Biplane ejection fraction (EF) of the LV was measured using the Simpsons Method from apical four-chamber (A4C) and apical two-chamber (A2C) views. The written TTE reports included information about paravalvular leakage (PVL), aortic regurgitation, pressure gradients, and effective orifice areas indexed (EOAI). Mean pressure gradient (MPG), proximal pressure gradient (PPG), acceleration time AT, and ejection time (ET) time were measured [22].

### 2.4. Surgical Technique

Each case was evaluated by the institutional Heart Team, while the final decision on the surgical technique and the applied bioprosthesis was made by the surgeon. The same group of highly qualified and experienced cardiac surgeons performed all procedures. After induction of anesthesia, intubation, and standard median sternotomy or ministernotomy, all patients were placed on CPB. Myocardial protection was achieved via antegrade administration of cold custodial cardioplegic solution for induction and was followed by antegrade and selective ostial administration of cold custodial cardioplegic solution, according to our institutional standard. Transverse aortotomy was performed approximately 1 cm above the sinotubular junction for both the Perceval and the Dokimos valve implantation, followed by resection of the native AV and the removal of the annular calcifications. A circumferential placement of annular sutures for conventional Dokimos implantation was performed, which was not needed for implantation of the Perceval as the sutureless implantation requires only three guiding sutures. Intraoperative evaluation of the prosthesis was applied using transesophageal echocardiography (TEE). All cases received the same standardized intraoperative and post-operative management.

### 2.5. Statistical Analysis

From a total of *n* = 272 patients, 201 were included in our final study cohort. Seventy-one patients (17 from the Perceval group and 54 from the Dokimos group) were excluded due to lack of data (*n* = 71). A test of normality was performed on all numerical variables using the Shapiro–Wilk test (*n* = 201). Numerical variables that followed a normal distribution were presented as mean ± standard deviation. Those that did not follow a normal distribution were presented as median (IQR). Independence *t*-test and Mann–Whitney U-test were used to compare the difference between the two groups according to the distribution followed. Comparison in each group for pre- and post-measurements was performed using the Wilcoxon test. Categorical variables were presented as frequencies and percentages (*n*, %). Chi-square test (X^2^) was used to compare the categorical variables associated with each group (Perceval, Dokimos). *p* values < 0.05 were considered statistically significant. All analyses were performed using the statistical software SPSS 22.0 (IBM Statistics).

The early hemodynamic and clinical outcomes of the two groups during the whole hospital stay were observed and compared. Primary endpoints were 30-day mortality, length of hospital stay, and pacemaker implantation. Secondary endpoints were echocardiographic parameters, complication rates, major adverse cardiovascular events, and prosthesis failure (grade II aortic regurgitation, paravalvular leak (PVL) with redo-intervention. Informed consent for treatment, data collection, and analysis was available. 

## 3. Results

### 3.1. Baseline, Procedural, and Clinical Characteristics

A total of 279 patients were screened, and 78 patients had to be excluded. The final cohort consisted of 201 patients. Of the 201 patients in the final cohort, *n* = 118 (58.7%) received a su-AVR using the Perceval bioprosthesis, while *n* = 83 (41.3%) received a conventional AVR with the Dokimos counterpart. The cohort’s mean age was 75.43 ± 6.12, and mean STS score 1.98 (1.48–2.92). The detailed characteristics are presented in Table 1 and Table 2. The two groups were well matched based on the pre-operative Lactate-Dehydrogenase (LDH) (Perceval vs. Dokimos: 217.0 (188.75–245.75) vs. 216.0 (191.0–249.0), *p* = 0.747) and Society of Thoracic Surgery risk of mortality (STS), (Perceval vs. Dokimos: 1.89 (1.48–2.4) vs. 2.07 (1.55–2.92), *p* = 0.083). Thus, there were no significant differences in LDH and STS risk of mortality pre-operatively between the two groups.

Mean total CPB time was 98.00 (79.00–126.00) minutes in the Perceval group and 111.34 (80.98–149.18) minutes in the Dokimos group (*p*: 0.081). The average ACC time was 62.5 (51.0–83.0) vs. 74.45 (53.32–102.00) minutes, respectively, *p* = 0.018. According to the mean ranks, patients in the Dokimos group had significantly longer ACC than those in the Perceval group. 

Six patients in the Perceval group and two in the Dokimos group died within the first 30 post-operative days (Pearson’s Chi-square X2 (1, N = 201) = 0.912, *p* value = 0.339 CI: (0.091–2.342). There were no significant differences between the two groups regarding 30 d mortality. 

During the intensive care unit (ICU) stay, cardiogenic shock occurred in *n* = 1 (0.8%) patient in the Perceval group. Re-do operation due to bleeding was needed in *n* = 8 (6.7%) patients from the Perceval group and in *n* = 9 (10.8%) from the Dokimos group. In both groups, no Extrakorporaler Life-Support (ECLS) was required after surgery. In both groups, a reduction in the total amount of thrombocytes and a rise in LDH levels occurred after surgery. There were no differences in post-operative drop of thrombocytes counts. However, the rise of LDH levels post-operatively was significantly higher in the Perceval group. The post-operative pacemaker implantation rate was similar in both groups. There were no significant differences in post-operative complications, such as patient prosthesis mismatch [23], MACCE, acute kidney injury (AKI), sepsis, and mortality. 

### 3.2. Echocardiographic Findings

Post-operatively, the mean pressure and the proximal pressure gradient in the Perceval group were significantly higher than in the Dokimos Group. Additionally, Perceval patients had significantly lower aortic valve area (AVA) compared to the Dokimos. No significant differences in post-operative parameters of left and right ventricular function, such as EF, were observed. 

No prosthesis-patient mismatch was observed in this cohort in the early post-operative period. Patients receiving Perceval showed an 8.4% incidence of (*n* = 10) mild PVL, and moderate PVL in *n* = 2 (1.7%), while only *n* = 2 (2.4%) patients showed a mild and *n* = 1 (1.2%) a severe PVL in Dokimos group (Table 3). 

## 4. Discussion

To our knowledge, this is one of the first studies that has been compared the performance of the Perceval sutureless bioprosthesis to its conventional counterpart, in this case, the Dokimus, and one of the few to specifically take hemodynamic results and overall clinical outcomes into consideration.

The key findings were that although the CPB and cross-clamp time in the Perceval group were indeed significantly reduced, as was expected due to the rapid deployment technology, the faster implantation had no impact on the primary endpoint of 30-day mortality. 

Hemodynamic parameters, i.e., MPG, PPG, were significantly higher in the Perceval than in the Dokimus group. No significant difference was confirmed concerning PVL between the groups. 

The sutureless valve techniques were developed with reported advantages over the conventional prosthesis, such as time-saving deployment with less manipulation, ease of implantation even in a minimally invasive approach, the ability of easy repositioning, shorter cross-clamp and CPB times, all of which collectively have been suggested to increase the suitability of this technique for elderly patients with multiple comorbidities [7,11,14,24,25]. In our study, we found no correlation between shorter cross-clamp and bypass times, on the one hand, and improved clinical outcomes, on the other, especially with respect to mortality, which is supported by other studies [23]. 

What we did find as a side observation is an increased rate of pneumonia and delirium in the Dokimus group. Whether this was related to the prolonged bypass and surgery and the ventilation time is controversial and is extensively discussed in the literature [16,26,27]. Another interesting observation was that despite increased rates of pneumonia and delirium in the Dokimus group, mean hospital stay in the Dokimus group was significantly shorter than in the Perceval group. This is in contrast to the findings of several studies reporting a shorter hospital stay for patients with sutureless valves compared to patients receiving conventional prostheses [17,27]. 

Regarding hemodynamic performance, we found increased transvalvular gradients in the Perceval group. To what degree these increased transvalvular gradients could be linked to stent deformation and the flutter phenomenon unique to the Perceval bioprosthesis described in the literature remains unclear [18,19].

In our study, no PPM, navigation, or migration of the prosthesis occurred. No significant differences were recorded in the incidence of high-grade paravalvular leakage.

At the beginning of the era of sutureless aortic valve replacement with Perceval, the Achilles heel of the valve was the increased rate of PM implantation post-operatively [28,29]. In our study, we found no significant differences in post-operative PM implantation rates between groups. This specific complication has been heavily discussed in the literature. In fact, several studies have found no difference in pacemaker implantation rates between both groups [13,17], while others found lower rates in pacemaker implantation post-operatively for patients who were implanted with the Perceval [26,27]. Post-operative pacemaker implantation rates depend on many factors, including surgeon experience, pre-existing (subclinical) conduction disorders, as well as the implantation technique itself, e.g., the extent of decalcification of the annulus, the placement of the guide sutures, and also the post-dilatation of the stent [30,31,32,33]. 

After considering all the results of our study, we have observed no clear advantage in the clinical outcomes of the sutureless prosthesis. Of course, there is also a randomized study that has proven that the sutureless prosthesis Perceval is not inferior to conventional prostheses [34]. The notable limitations of this study are the retrospective and single-center nature, as well as the small cohort number. Moreover, although the two groups were well matched based on their STS mortality risk score, which takes age into account in each case, the sutureless group still consists of older patients when compared to the conventional group. This age difference could be considered as a limitation of our study. There are no other studies comparing Perceval with Dokimus bioprosthesis to have as a reference. Moreover, we only report the early post-operative findings of the patients to the time of their discharge and 30-days mortality. Long-term durability is of major importance from the patient perspective and, therefore, warrants close consideration by the entire team involved in the management of patients with severe aortic valve stenosis.

## 5. Conclusions

Regarding the clinical outcomes, Perceval was equivalent and not superior to the Dokimus bioprosthesis. Instead, the Perceval prosthesis resulted in higher gradients and longer hospital stays. The modern medical era thrives on new technologies, and sutureless technology is an extension of conventional aortic valve replacement. However, despite the recent sutureless developments, there are still controversial data and challenges to overcome. Thus, the suitability of the Perceval prosthesis implantation must be discussed on a case-by-case basis. In the future, larger and possibly prospective reports of different models of conventional bioprosthesis in relation to the Perceval alternative regarding the short-term, mid-term, and long-term clinical outcomes are needed to confirm these findings and demonstrate the potential clinical advantage of these valves.

## Figures and Tables

**Table 1:** Demographic, pre-operative, and operative characteristics.

Variable	Perceval (*n* = 118)	Dokimos (*n* = 83)	*p*-Value
Age (years)	76.26 ± 5.62	73.54 ± 6.33	<0.001
Male gender, *n* (%)	51 (43.20)	53 (63.80)	0.041
BMI kg/m^2^	27.50 (24.30–30.8)	25.91 (24.22–29.67)	0.060
COPD, *n* (%)	10 (8.4)	9 (10.80)	0.556
NYHA > II	17 (14.4)	23 (27.70)	0.02
STS mortality risk (%)	1.89 (1.48–2.4)	2.07 (1.55–2.92)	0.083
LDH pre-op	217.00 (188.75–245.75)	216.00 (191.00–249.00)	0.747
Thrombocytes pre-op (×10^3^ µL)	244.50 (204.75–284.0)	213.0 (150.00–265.00)	0.005
Cross-clamp time in minutes	62.50 (51.00–83.00)	74.45 (53.32–102.00)	0.018
CPB Time in minutes	98.00 (79.00–126.00)	111.34 (80.98–149.18)	0.081
LDH post-op	332.0 (283.00–382.00)	217 (188.75–244.25)	<0.001
Thrombocytes post-op (×10^3^ µL)	124.5 (102.75–165.50)	121.0 (95.00–146.00)	0.130

BMI: Body mass index; COPD: Chronic obstructive pulmonary disease; NYHA: New York Heart Association functional class; STS: Society of Thoracic Surgeons, LDH: Lactate dehydrogenase, CPB: Cardiopulmonary bypass.

**Table 2:** Post-operative complications and clinical outcomes.

Variable	Perceval (*n* = 118)	Dokimos (*n* = 83)	*p* Value
AV-block with PM implantation need, *n* (%)	10 (8.4)	9 (10.8)	0.556
VHF, *n* (%)	30 (25.4)	26 (31.3)	0.359
Other arrhythmias, *n* (%)	7 (6.0)	3 (3.6)	0.443
Bleeding with Re-do, *n* (%)	8 (6.7)	9 (10.8)	0.302
Pneumonia, *n* (%)	15 (12.7)	28 (33.7)	<0.001
Delir, *n* (%)	14 (11.8)	25 (30.1)	<0.001
Pleural effusion, *n* (%)	11 (9.3)	8 (9.6)	0.943
Apoplex, *n* (%)	1 (0.8)	0 (0.0)	0.415
CPR, *n* (%)	1 (0.8)	0 (0.0)	0.415
AKNI, *n* (%)	2 (1.6)	7 (8.4)	0.021
Cardiogenic Shock, *n* (%)	1 (0.8)	0 (0.0)	0.415
Significant pericardial effusion, *n* (%)	1 (0.8)	0 (0.0)	0.415
HIT, *n* (%)	0 (0.0)	0 (0.0)	-
Sepsis, *n* (%)	1 (0.8)	0 (0.0)	0.415
Mild PVL, *n* (%)	10 (8.4)	2 (2.4)	0.075
Moderate PVL, *n* (%)	2 (1.7)	0 (0.0)	0.233
Severe PVL, *n* (%)	0 (0.0)	1 (1.2)	0.234
PPM, *n* (%)	0 (0.0)	0 (0.0)	-
Valve Dislocation, Navigation	0 (0.0)	0 (0.0)	-
Need of ECLS	0 (0.0)	0 (0.0)	-
30 days Mortality, *n* (%)	6 (5.3)	2 (2.4)	0.339
Hospital stay (d)	17.00 (13.00–27.00)	13.00 (9.00–17.00)	<0.001

PM: Pacemaker, HIT: Heparin-induced thrombocytopenia, PVL: Paravalvular leakage, PPM: Patient prosthesis mismatch, ECLS: Extracorporeal life support.

**Table 3:** Post-operative echocardiographic findings.

Variable	Perceval (*n* = 118)	Dokimos (*n* = 83)	*p* Value
MPG	12.12 (10.0–17.0)	9.0 (7.00–13.0)	<0.001
PPG	24.0 (19.00–30.00)	18.0 (14.00–25.00)	<0.001
AVA (Vmax)	1.10 (0.9–1.39)	1.72 (1.39–2.10)	<0.001
EOAI (VTI)	0.65 (0.5–0.79)	0.96 (0.79–1.20)	<0.001
EOAI (Vmax)	0.61 (0.49–0.77)	0.94 (0.78–1.16)	<0.001
ET	255.30 ± 32.86	266.9 ± 32.35	0.028
AT	66.41 ± 18.31	69.89 ± 15.06	0.200
EF %	55.00 (50.00–58.00)	56.00 (50.00–58.00)	0.144
TAPSE	15.00 (13.00–17.00)	14.3 (13.20–16.10)	0.396
IVSd	14.00 (13.1–16.00)	14.25 (13.2–16.15)	0.019
LVSV	53.00 (42.25–65.5)	65.00 (55.00–82.00)	<0.001
Velocity ratio	0.42 (0.38–0.49)	0.53 (0.44–0.62)	<0.001

Mean ± SD, median (IQR), *n* (%), MPG: Mean pressure gradient, PPG: Peak pressure gradient, LVSV: Left ventricle stroke volume, AVAVmax: Peak aortic jet velocity, EOAI(VTI): Effective orifice area indexed to the body surface area (velocity time integral), EOAI (Vmax): Effective orifice area indexed to the body surface area (velocity max). AT: Acceleration time, ET: Ejection time, EF: Ejection fraction, TAPSE: Tricuspid annular plane systolic excursion, IVSd: Intraventricular septum diameter.

## Data Availability

All data are available at the University Hospital Aachen, covered by Ali Aljalloud.
